# Influence of focus of attention, reinvestment and fall history on elderly gait stability

**DOI:** 10.14814/phy2.13061

**Published:** 2017-01-11

**Authors:** Jonathan L. A. de Melker Worms, John F. Stins, Erwin E. H. van Wegen, Ian D. Loram, Peter J. Beek

**Affiliations:** ^1^ Department of Human Movement SciencesFaculty of Behavioural and Movement SciencesVrije Universiteit AmsterdamMOVE Research Institute AmsterdamAmsterdamthe Netherlands; ^2^ Cognitive Motor Function research groupSchool of Healthcare ScienceManchester Metropolitan UniversityManchesterUnited Kingdom; ^3^ Department of Rehabilitation MedicineVU University Medical CenterMOVE Research Institute AmsterdamAmsterdamthe Netherlands

**Keywords:** Attentional focus, elderly, falls, gait, motion capture

## Abstract

Falls represent a substantial risk in the elderly. Previous studies have found that a focus on the outcome or effect of the movement (external focus of attention) leads to improved balance performance, whereas a focus on the movement execution itself (internal focus of attention) impairs balance performance in elderly. A shift toward more conscious, explicit forms of motor control occurs when existing declarative knowledge is recruited in motor control, a phenomenon called reinvestment. We investigated the effects of attentional focus and reinvestment on gait stability in elderly fallers and nonfallers. Full body kinematics was collected from twenty‐eight healthy older adults walking on a treadmill, while focus of attention was manipulated through instruction. Participants also filled out the Movement Specific Reinvestment Scale (MSRS) and the Falls Efficacy Scale International (FES‐I), and provided details about their fall history. Coefficients of Variation (CV) of spatiotemporal gait parameters and Local Divergence Exponents (LDE) were calculated as measures of gait variability and gait stability, respectively. Larger stance time CV and LDE (decreased gait stability) were found for fallers compared to nonfallers. No significant effect of attentional focus was found for the gait parameters, and no significant relation between MSRS score (reinvestment) and fall history was found. We conclude that external attention to the walking surface does not lead to improved gait stability in elderly. Potential benefits of an external focus of attention might not apply to gait, because walking movements are not geared toward achieving a distinct environmental effect.

## Introduction

In the elderly population, falls represent a substantial risk. Approximately, two‐thirds of unintentional injury‐related deaths in older adults are caused by falls (Baker and Harvey 1985). Falls represent the leading cause of bone fractures (Schwartz et al. 2005) and one‐third of community‐dwelling elderly over the age of 65 suffer at least one fall each year. Consequently, falls impose a substantial global economic burden (Stevens et al. 2006).

There is considerable interest in psychological/cognitive factors that determine gait performance, and hence fall risk. In pertinent literature, it has been suggested that fall risk is larger for individuals with a higher level of conscious attention to their own movements than the general population (Wong et al. 2008; Chiviacowsky et al. 2010; Wulf 2013; Young et al. 2016). It has further been suggested that the fall risk of such individuals might be reduced if their movements would be more automated and thus require less attentional control (Chiviacowsky et al. 2010; Wulf 2013; Young et al. 2016). Conversely, shifts toward more conscious, explicit forms of motor control occur when existing declarative knowledge is recruited in the planning and execution of movements. Masters et al. (1993) dubbed this phenomenon reinvestment (i.e., of the aforementioned declarative knowledge). Reinvestment is thought to be manifested when an individual is highly motivated or under pressure, or has difficulty to move successfully (Wong et al. 2008). Using the Movement Specific Reinvestment Scale (MSRS), Wong et al. (2008) found that elderly with a history of falling had a higher predisposition to reinvest compared to elderly nonfallers.

Allied to the theory of reinvestment is the ‘constrained action hypothesis’ (Wulf and Prinz 2001), which emphasizes the crucial role of attentional processes in motor performance. By now, there is ample evidence that an attentional focus on the outcome/effect of the movement (‘external focus of attention’) leads to improved motor performance and learning, whereas a focus on the movement execution itself (‘internal focus of attention’) hampers motor performance and learning. A review by Wulf (2013) showed that these effects have been found for a wide range of sports and balancing tasks. Chiviacowsky et al. (2010) showed that this effect generalizes to motor learning of balance control in the elderly population, using an unstable balance board to assess balance performance. In a recent study linking the concept of attentional focus to that of reinvestment, higher reinvestment was found to be suggestive of a preference for an internally directed attentional focus (Kal et al. 2015). According to the constrained action hypothesis (Wulf and Prinz 2001), an internal focus of attention induces a conscious control of movement that impairs automaticity. Moreover, this theory states that an external focus of attention enhances automaticity and allows for more efficient, implicit control mechanisms to come into play. In subsequent papers, this claim of enhanced automaticity received empirical support in the form of reduced muscular activity (Zachry et al. 2005; Lohse et al. 2010), and more fluent and more regular movement (Kal et al. 2013).

In some tasks, the goal is not so much to achieve a particular environmental effect, as in goal‐directed instrumental actions, but rather to control the movements of the body itself. In such instances, an external focus of attention might be induced by directing attention to physical surfaces in the environment on which forces are exerted through muscle activity, such as the ground one is standing on in a gymnastics floor routine (Lawrence et al. 2011). Critical for the proper use of the term external focus of attention in such situations is not only that reference is made to physical properties of the environment, but also that this reference is relevant to successful performance of the task (Lawrence et al. 2011; An et al. 2013).

Even though benefits of an external focus of attention have been found for postural balance control, such benefits have to date not been established for elderly balance in gait. In this study, we therefore investigated the effects of attentional focus (a state variable) and reinvestment (a trait variable) on gait stability and variability in elderly fallers and nonfallers.

The literature on the relation between elderly fallers and gait performance shows that gait variability is increased in elderly fallers compared to nonfallers (Hausdorff et al. 1997; Toebes et al. 2012). Furthermore, prospective research has shown an increased fall risk for elderly with increased stride‐to‐stride gait variability (Hausdorff et al. 2001). An alternative approach to assess gait performance is through gait stability, which has been quantified using Local Divergence Exponents (LDE) (Rosenstein et al. 1993; Liu et al. 2008; Lockhart and Liu 2008; Toebes et al. 2012). The gait of elderly fallers has been shown to be less stable than that of nonfallers in terms of such LDE values (Liu et al. 2008; Lockhart and Liu 2008; Toebes et al. 2012).

We assessed whether an external focus of attention leads to a more stable walking pattern and reduced gait variability compared to an internal focus of attention. We further investigated how fall history, concern about falling, and reinvestment interact with the gait stability parameters, and whether fall history affects concern about falling, reinvestment, and gait stability. To this end, we calculated coefficients of variation (CVs) of step length, step width, stance time, and swing time, as measures of gait variability. In addition, we calculated LDE values for the Centre of Mass (CoM) velocity time series (Rosenstein et al. 1993), as a measure of gait stability. We expected increased gait stability and reduced gait variability for the external focus condition compared to the internal focus condition.

## Method

### Participants

Twenty‐eight healthy older adults (eight males, 20 females, age: 65 + years) were recruited, with an average participant age of 69.3 ± 3.7 years (mean ± standard deviation; range: 65–78). A Dutch version of the Mini‐Mental State Examination (MMSE) was used to determine the cognitive status of participants, and they had to be able to walk independently for 10 min without a walking aid. Participants with a MMSE score below 25/30, any history of rheumatoid arthritis in lower extremities, cerebral vascular disease, Parkinson's disease, peripheral neuropathy, cardiac arrest, bypass treatment, or any other neurological, or cardiovascular impairment were excluded from the study. The study received approval from the local ethical committee and participants gave written informed consent prior to their participation.

### Material

Gait performance can be assessed by measurement of either steady‐state gait or perturbed walking. Investigation of perturbed walking involves analysis of the manner in which the actor attempts to regain stability following a perturbation (Bruijn et al. 2010; Granacher et al. 2010). In this study, we adopted a paradigm involving transient mechanical perturbations. The perturbations consisted of unilateral decelerations of a split‐belt treadmill, which led to a forward slip of the foot, as when walking on a slippery surface. The perturbations in question were applied at unexpected moments during the walking bouts. Therefore, the threat of a perturbation and the participants' motivation to preserve a stable locomotion pattern were present throughout the whole trial. We here focus on steady gait performance in between the stabilizing responses to the perturbations. The direct stabilizing responses within the first 4 sec after each perturbation will be reported in a separate paper because each of the two modes of gait assessment brings along specific theoretical and methodological issues. In order to examine how attentional focus and reinvestment scores affect gait stability, we collected full body kinematics and analyzed participants' steady gait bouts between the balance recovery responses to the perturbations.

**Figure 1:**
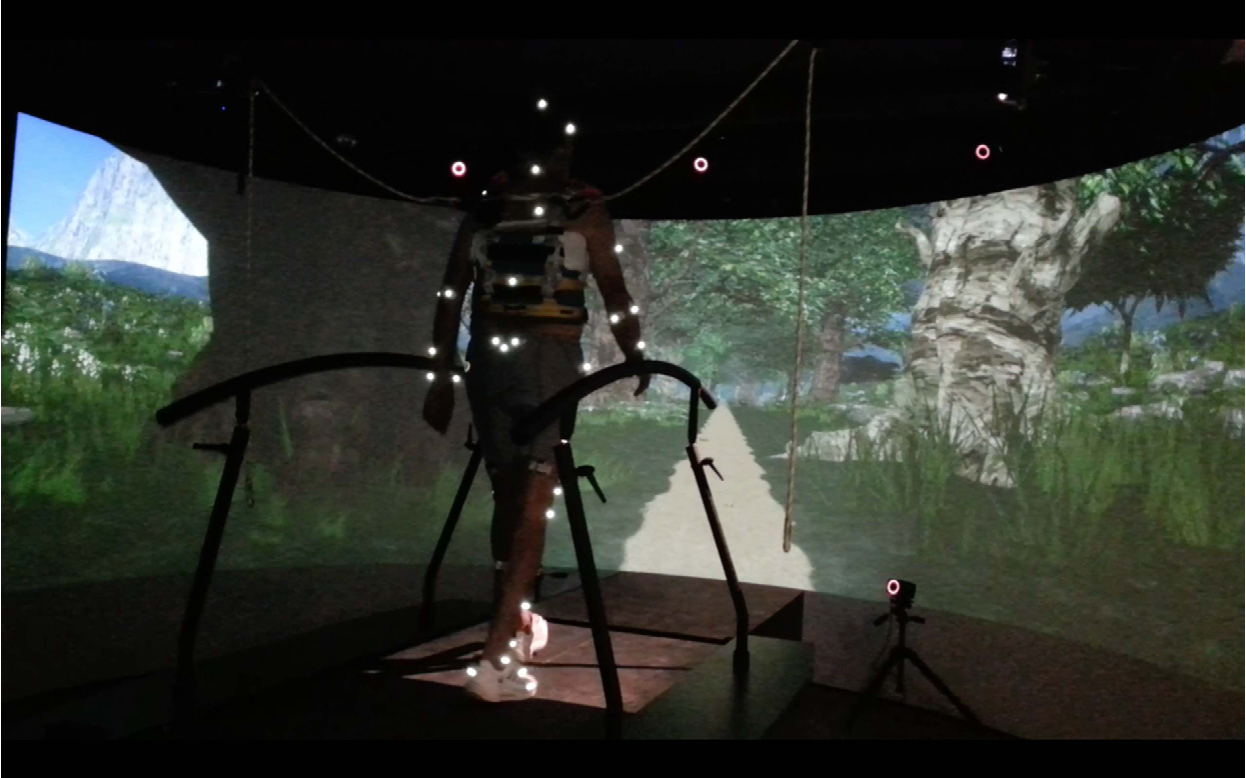
Virtual walking environment.

Participants walked on a split‐belt treadmill at a fixed speed of 1 m/sec with a 180 degrees semicircular screen in front of them (GRAIL system, Motekforce Link b.v., Amsterdam, The Netherlands). A realistic optical flow pattern, coupled to the treadmill velocity, was projected on the screen and displayed a straight forest road with mountains (Fig. 1). The participants' gait was occasionally perturbed through transient unilateral treadmill decelerations that were initiated right after toe off of the dominant leg. At the following heel strike, the velocity of this half of the treadmill was reduced to 0 m/sec, causing a gait perturbation. At the next heel strike of the dominant leg, the treadmill belt had regained its original velocity of 1 m/sec. The perturbations were experienced as a forward slip of the foot. The system was controlled, using D‐Flow software from Motekforce Link b.v. Full body kinematics was collected using 47 passive retroreflective markers (using the Human Body Model from Motekforce Link b.v. (van den Bogert et al. 2013)) and 10 high‐resolution infrared cameras (Vicon, Oxford, UK).

### Questionnaires

Before the experiment, reinvestment propensity was assessed, using a Dutch version of the Movement Specific Reinvestment Scale (MSRS) (Kleynen et al. 2013). Additionally, a Dutch version of the Falls Efficacy Scale International (FES‐I) (Kempen et al. 2007) was completed and fall history details were collected. We defined a fall as follows: “An event in which a person unintentionally comes to rest on the ground or other lower levels” (Gibson et al. 1987; de Zwart et al. 2015). Falls that resulted from loss of consciousness or acute paralysis caused by stroke, epileptic attacks or violence were not included. When one or more falls had occurred within 12 months prior to the experiment, participants were labeled as fallers. The other participants were labelled as nonfallers.

The FES‐I is a measure quantifying an individual's concern about falling during various tasks (Morgan et al. 2013; Visschedijk et al. 2015), yielding a score between 16 (low concern about falling) and 64 (high concern about falling). The MSRS is a measure of an individual's propensity for reinvestment and consists of two subscales, pertaining to conscious motor processing (CMP) and movement self‐consciousness (MSC), respectively. The first subscale is related to the amount of conscious monitoring of one's own movement, whereas the latter is related to the amount of concern, as related to movement (Wong et al. 2008).

### Procedure

A fixed walking speed of 1 m/sec (i.e., 3.6 km/h) was used throughout the experiment, gait perturbations excluded. Participants were first familiarized with 5 min of treadmill walking including gait perturbations. This was followed by two bouts of 5 min of walking, one with an internal focus of intention instruction and one with an external focus of attention instruction, conducted in counterbalanced order. In the internal focus of attention condition, participants were instructed to look ahead at the screen and concentrate on the movement of their legs. In the external focus of attention condition, they were instructed to look ahead at the screen and concentrate on the movement of the treadmill belt. The instructions were repeated through a speaker system every 30 sec. For each condition, 20 perturbations were given at heel strike, at random time intervals varying between 10 and 20 sec. As this experiment was part of a protocol involving multiple experiments, participants had already walked on the treadmill for 20 min at the start of the experiment.

### Spatiotemporal gait parameters

From the focus of attention trials, the sections of unperturbed gait between 4 sec after each perturbation up until the next perturbation were analyzed. From these gait bouts (ranging from 6 to 16 sec in duration), we calculated the participants' means and CV of the following spatiotemporal gait parameters for the dominant leg: step length, step width, stance time, and swing time.

Step length was calculated as the distance in the anterior‐posterior direction between the toe marker of the nondominant leg and heel marker of the dominant leg, at each heel strike of the dominant leg. Step width was calculated as the distance between the toe marker of the dominant leg and the toe marker of the nondominant leg in the mediolateral direction, at each heel strike of the dominant leg. Stance time was defined as the time interval between heel strike and toe off, while swing time was defined as the time interval between toe off and heel strike. Per participant, the CV of these spatiotemporal gait parameters was calculated according to equation 1.

(1) $$\text{CV}\left(\%\right)=100\times \frac{\text{standard deviation}}{\text{mean}}$$

#### Local divergence exponents (LDE)

The LDE is a measure derived from dynamical system theory, and refers to the sensitivity of a system to perturbations. Lower LDE values correspond with increased gait stability (Bruijn et al. 2012). LDE was calculated for the three dimensions of the COM velocity signals. A state space reconstruction in nine dimensions was used, including two time delayed copies of the three COM velocity dimensions, one with 10 samples (0.1 sec) and one with 20 samples (0.2 sec) time delay (van Schooten et al. 2013). Rosenstein's algorithm was employed to track the average logarithmic divergence between neighboring trajectories in the reconstructed state space (Rosenstein et al. 1993). LDE was quantified as the slope of the first 60 samples (0.6 sec) of the divergence curve, which roughly corresponded to one step, and was calculated over equal‐length time series of 7 sec. All calculations were implemented in Matlab (version R2014a, The MathWorks, Inc., Natick, MA).

### Statistical analysis

All dependent variables were tested for normality, using the Shapiro–Wilk test. For the variables that did not pass the test we used nonparametric tests.

To study effects on the questionnaire data by the occurrence of a fall in the past 12 months, FES‐I, CMP and MSC scores of fallers were compared to nonfallers, using Mann–Whitney U tests, effect size (r) and Bayes factors. Additionally, correlations between all gait parameters (mean and CV of spatiotemporal gait parameters and LDE) versus the questionnaires (FES‐I, MSRS CMP and MSRS MSC) were calculated, using Spearman's Rho.

A 2 *×* 2 mixed ANOVA (within and between subjects) was used to test whether participant means of the normally distributed gait parameters (step length, step width, stance time, swing time and LDE) were significantly different between the focus of attention conditions, between fallers and nonfallers, and whether significant interaction effects were present between fall history and attention. The CVs of the spatiotemporal gait parameters did not pass the Shapiro–Wilk test for normality. Effects of fall history on these variables were calculated, using Mann–Whitney U tests. Bonferroni corrections were used for subsequent Mann–Whitney U tests for fall history effects within attention conditions. Wilcoxon signed‐rank tests were used to calculate effects of internal versus external attention. Bonferroni corrections were used for subsequent Wilcoxon signed‐rank tests for attention condition effects within fallers and nonfallers.

In addition to the above tests for significance, we calculated effect size and employed Bayesian statistics, allowing us to calculate the Bayes factor. The Bayes factor (BF_10_) represents the likelihood of the alternative hypothesis versus the null hypothesis. Bayes factors can be used to accept the null hypothesis, which is impossible on the basis of just *P*‐values. It has been recommended to label BF_10_ values lower than 0.3 as moderate evidence in favor of the null hypothesis, and higher than 3.0 as moderate evidence in favor of the alternative hypothesis (Lee and Wagenmakers 2014). All statistical analysis was done using IBM SPSS Statistics 20.0, except for the Bayes factors which were calculated with the BayesFactor v0.9.12‐2 package for R (bayesfactorpcl.r‐forge.r‐project.org; R‐project.org).

## Results

### Fall history, concern about falling and reinvestment

Nine out of twenty‐eight participants had experienced a fall within the last 12 months and were labeled as fallers (age: 70.44 ± 3.69, BMI: 25.4 ± 2.47), while the remaining participants were labeled as nonfallers (age: 69.37 ± 3.65, BMI: 25.2 ± 2.39). The higher FES‐I score for fallers than for nonfallers was borderline significant (Table 1). The CMP and MSC scores on the MSRS were not significantly different between fallers and nonfallers. Furthermore, no significant correlation was found between any of the gait parameters versus any of the questionnaires (FES‐I, MSRS CMP and MSRS MSC).

**Table 1:** Fallers and nonfallers compared. Means (standard deviation), *P*‐values, effect sizes and Bayes factors (BF_10_) are shown for the tested gait parameters

	Fallers	Nonfallers	*P*‐value	Effect size	Bayes factor
Mean step length (mm)	508 (70)	552 (50)	0.07	*η* ^*2*^ = 0.12	1.33
Mean step width (mm)	147 (35)	134 (29)	0.30	*η* ^*2*^ = 0.04	0.55
Mean stance time (s)	0.69 (0.09)	0.73 (0.06)	0.20	*η* ^*2*^ = 0.06	0.68
Mean swing time (s)	0.38 (0.03)	0.41 (0.03)	0.09	*η* ^*2*^ = 0.11	1.12
CV step length (%)	4.50 (1.21)	4.24 (1.44)	0.29	*r *=* *0.20	0.40
CV step width (%)	15.61 (5.96)	18.59 (5.67)	0.07	*r *=* *0.34	0.67
CV stance time (%)	3.50 (0.56)	3.01 (0.75)	**0.02**	*r *=* *0.46	1.05
CV swing time (%)	4.94 (1.50)	4.41 (1.18)	0.32	*r *=* *0.22	0.53
LDE	0.97 (0.12)	0.88 (0.08)	**0.03**	*η* ^*2*^ = 0.16	2.20
FES‐I	20 (6)	17 (3)	0.06	*r *=* *0.37	1.39
MSRS ‐ CMP	8 (8)	12 (12.5)	0.64	*r *=* *0.09	0.43
MSRS – MSC	5 (5)	6 (6)	0.47	*r *=* *0.14	0.42

Only for the CV, FES‐I and MSRS variables medians (interquartile range) are given. The Bayes factor (BF_10_) indicates the odds for the alternative hypothesis versus the null hypothesis to be true. Significant p‐values are displayed in bold.

LDE, local divergence exponent (gait stability); CV, coefficient of variation; CMP, conscious motor processing; MSC, movement self‐consciousness; MSRS, Movement Specific Reinvestment Scale.

### Gait parameters

**Figure 2:**
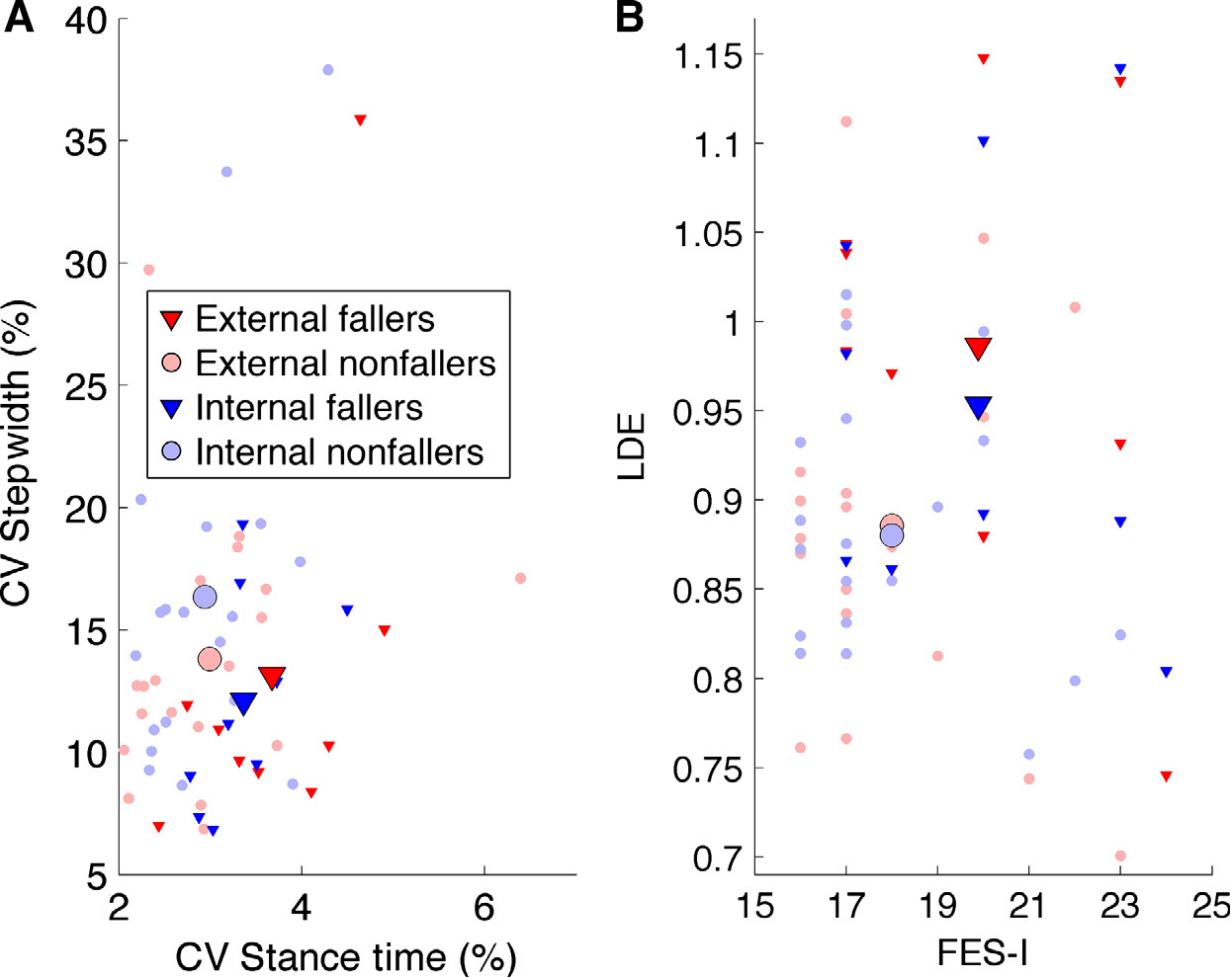
(A) Stance time and step width variability (CV) are shown for each participant in both attention conditions. Fallers had significantly higher stance time CV, and the lower step width CV compared to nonfallers was borderline significant. No significant difference was found between internal or external attention for any of the gait parameters. (B) Local divergence exponents (LDE) and FES‐I are shown for each participant in both attention conditions. Fallers had significantly higher LDE values (lower gait stability) than nonfallers. The higher FES‐I score for fallers than nonfallers was borderline significant. Between internal and external attention no significant difference was found for FES‐I or LDE. CV, Coefficient of Variation.

For fallers, the stance time CV and LDE were significantly larger than for nonfallers, however Bayes factor analysis did not provide evidence for this difference. The larger FES‐I score, smaller step width CV and smaller step length for fallers compared to nonfallers was borderline significant (Fig. 2 & Table 1). No significant differences were found between the internal focus of attention condition and the external focus of attention condition for any of the gait parameters (Table 2). Furthermore, no significant interaction effects were found.

**Table 2:** The internal and external attention conditions compared. Means (standard deviation), *P*‐values, effect sizes and Bayes factors are shown for the tested gait parameters

	Internal focus	External focus	*P*‐value	Effect size	Bayes Factor
Mean step length (mm)	536 (58)	540 (62)	0.32	*η* ^*2*^ = 0.04	0.33
Mean step width (mm)	136 (32)	140 (31)	0.14	*η* ^*2*^ = 0.08	0.87
Mean stance time (s)	0.71 (0.07)	0.72 (0.07)	0.11	*η* ^*2*^ = 0.10	0.67
Mean swing time (s)	0.40 (0.03)	0.40 (0.03)	0.91	*η* ^*2*^ = 0.00	**0.27**
CV step length (%)	4.23 (1.18)	4.42 (1.62)	0.35	*r *=* *0.18	0.39
CV step width (%)	18.51 (7.29)	16.75 (5.71)	0.09	*r *=* *0.32	0.59
CV stance time (%)	3.17 (0.63)	3.16 (0.99)	0.84	*r *=* *0.04	**0.20**
CV swing time (%)	4.57 (1.24)	4.60 (1.55)	0.91	*r *=* *0.02	**0.20**
LDE	0.92 (0.12)	0.90 (0.09)	0.21	*η* ^*2*^ = 0.06	0.35

Only for the CV variables medians (interquartile range) are given. The Bayes factor (BF_10_) indicates the odds for the alternative hypothesis vs. the null hypothesis to be true. Bayes factors smaller than 1/3 are shown in bold.

LDE, local divergence exponent (gait stability); CV, coefficient of variation.

## Discussion

In this study, we investigated whether an external focus of attention temporarily increases gait stability and/or decreases gait variability compared to an internal focus of attention. The higher LDE and stance time CV indicated significantly lower gait stability and increased gait variability for fallers compared to nonfallers. These results support previous suggestions that gait stability (Liu et al. 2008; Lockhart and Liu 2008; Toebes et al. 2012) and gait variability (Hausdorff et al. 1997; Toebes et al. 2012) are associated with fall history. However, no significant effect of attentional focus was found for any of the gait parameters. Furthermore, Bayesian analysis provided moderate evidence for the null hypothesis that attentional focus does not affect gait variability, based on the CVs of stance time and swing time.

The results might also suggest higher falls efficacy for fallers compared to nonfallers, as the effect of fall history on FES‐I score was borderline significant. However, no significant effect of fall history on the MSRS reinvestment scores was found for either the CMP or MSC subscales. Thus, having experienced falls was not associated with increased reinvestment, which seems to be in contrast to findings from Wong et al. (2008), who found increased MSRS scores for elderly fallers. On the other hand, the Bayes factors did not provide evidence to accept the null hypothesis that fall history does not affect reinvestment. A possible limitation is the relatively low sample size of participants that experienced a fall (nine) compared to the number of nonfallers (seventeen).

### Effect of visual feedback

In a recent review on the effects of internal and external focus of attention on motor performance (Wulf 2013), several other studies were evaluated where null effects of attentional focus were found as well. For some of these studies, participants were presented with information on a screen about their movements or the effects of their movements in the environment (De Bruin et al. 2009; Shafizadeh et al. 2013). For example, a moving dot representing the center of gravity relative to a target (De Bruin et al. 2009). Wulf (2013) argued that null effects in these studies were caused by powerful visual feedback, which presumably obfuscated attentional focus effects. For our study, participants received visual information of realistic optic flow that is perceived with normal gait. Therefore, one might also attribute our null effect to the presence of powerful visual feedback. As such, it might be possible that the presented optic flow overruled the effects of the instructions to concentrate on the movements of the treadmill or legs. However, there is reason to believe that effects of attentional focus can still manifest themselves in the presence of powerful visual feedback. It is well established that visual information of the surroundings aids to determine one's location in space and bodily orientation. This visual feedback is powerful, for example, as balancing on an unstable surface (e.g., stabilometer or balance disk) with the eyes closed is much more challenging than with eyes open. Nevertheless, for multiple balancing experiments, effects of attentional focus were found (Wulf et al. 1998, 2001, 2004, 2007; Shea and Wulf 1999; McNevin et al. 2003; Wulf and McNevin 2003; Chiviacowsky et al. 2010). These attentional focus effects occurred while participants had their eyes open and were highly dependent on the visual information to regulate their balance. Therefore, the powerful visual feedback did not obfuscate attentional focus effects for these studies. As such, it also seems unlikely that the optic flow one perceives with gait obfuscates attentional focus effects on gait performance.

### Evaluating effects of attentional focus and reinvestment on gait stability

The results of this study could suggest that benefits of an external focus of attention are only present when the instructions imply a movement task originated by the performer, that is, the direct effect of the movement. Moreover, in the literature on attentional focus, most studies involved a task in which actors were instructed to achieve a specific environmental effect. In that case, an external focus of attention could provide information that facilitates smooth planning and execution of the instrumental actions required to achieve that effect. However, the task considered in the present experiment was to control movement of the body itself (i.e., locomotion), in the absence of a distinct environmental goal. In other studies where the task was to control body movement without such a goal, results have been equivocal; for example, improved swimming performance for an external focus instruction compared to an internal focus instruction was found (Freudenheim et al. 2010; Stoate and Wulf 2011), while no attentional focus effect was found by Lawrence et al. (2011) who compared internal and external focus on motor learning for a gymnastics floor routine. Moreover, Kal et al. (2015) even suggested an opposite effect, whereby external focus in fact reduced automaticity of paretic leg movement of stroke patients. As such, some authors argued that benefits of an external focus of attention do not apply to motor tasks where performance only depends on the movement form or movement pattern of the body itself, and where movement effects on the environment are not of main importance (Lawrence et al. 2011; Peh et al. 2011). Subsequently, Wulf (2013) criticized this view by arguing that the instructions adopted in the gymnastics study (Lawrence et al. 2011) were not relevant to performance of the gymnastics task. Furthermore, multiple other studies did show improvements in movement form (kinematics) with an external focus of attention, for example for golf swing (An et al. 2013), darts throwing (Lohse et al. 2010), rowing (Parr and Button 2009) and ball throwing (Southard 2011). However, for all of these studies, manipulation of an external object was involved and the effect of the movement in the environment was integral to the task.

According to the theory of reinvestment, a reduced falls efficacy or increased fear of falling could lead to increased conscious attention to movement of the body. This could interfere with the automaticity of motor control and revert the actor back to an earlier declarative stage of learning. Analogous to reinvestment theory, an internal focus of attention might trigger the same adverse process. This might explain reduced performance with an internal focus of attention compared to an external focus of attention in ontogenic skills (learned later in life), for example, with postural control on a stabilometer (Chiviacowsky et al. 2010) and with various sports (Freudenheim et al. 2010; Lohse et al. 2010; Wulf et al. 2010). However, because walking and normal postural control on solid ground are phylogenic skills (learned in early childhood, without declarative knowledge), it is unlikely that an internal focus of attention could lead to such a reversal (Young and Williams 2015). This was supported by findings in postural balance control while standing on solid ground, where no benefits of an external focus of attention over an internal focus of attention were found (Wulf et al. 2007). It should be noted that two previous studies involving patients did find an effect of attentional focus on gait performance (Canning 2005; Shafizadeh et al. 2013). Canning (2005) found improved gait performance for an internal focus of attention instead of an external focus of attention in Parkinson's disease patients. Gait performance was assessed while participants carried a tray with glasses. Attention was either directed to walking (internal focus) or to balancing the tray with glasses (external focus). However, one could argue that in this experiment a focus on two different aspects of the task was compared, while performance of only one of those aspects was assessed (Wulf 2013). Therefore, the inferred benefit of an internal focus of attention might be challenged. Shafizadeh et al. (2013) found an effect of improved gait performance for an external focus of attention in multiple sclerosis patients compared to an internal focus of attention. However, in their experimental conditions, different modes of gait performance feedback were used to focus attention. In the internal focus of attention condition, different information of gait parameters was presented on a screen than in the external focus of attention condition, in which auditory feedback was added as well. Therefore, in that study, the observed effect on gait performance could be caused by the inequality of information that was given, as opposed to a cause of attentional focus.

This study adds to the growing body of literature on the effects of reinvestment and attentional focus on gait stability in elderly and the interaction with fall history. We found that these psychological/cognitive factors had little effect on gait performance. A general limitation with studies manipulating attentional focus using verbal instruction is that it is not possible to independently assess whether participants complied with the instructions. We tried to undercut this drawback by repeating the instructions every 30 sec, but still there was no guarantee that attentional focus was successfully manipulated. In previous studies on balance control, the effects of attentional focus were only found when balance was challenged, for example, when using an unstable standing surface (Wulf et al. 1998, 2001, 2004, 2007; Shea and Wulf 1999; McNevin et al. 2003; Wulf and McNevin 2003; Chiviacowsky et al. 2010), but not for normal standing (Wulf et al. 2007). Perhaps the effects of attentional focus could also emerge for walking when the task to maintain a particular walking pattern would be more challenging, for example, through continuous gait perturbations. A possible limitation of this study is that the expectation of a perturbation affected the gait pattern of the unperturbed walking bouts between the perturbations. In other words, it might be difficult to directly compare our gait data to normal unperturbed walking. On the other hand, threats of gait perturbations in real life would also alter one's gait pattern, for example, when walking on a slippery road. In such instances heightened awareness of imminent fall risk may interfere with maintaining a normal (and automatized) gait pattern. In addition, it might be possible that there are motor learning effects of attentional focus on walking performance, but no acute effects. In that case, the addition of retention tests might also reveal a relation between gait performance and attentional focus. Further investigation of this topic could also clarify whether external attention instructions remain problematic in tasks where one does not move or manipulate an external object.

## Conclusions

The results of this study provide further insight into the relations between gait variability, gait stability and falls in the elderly, based on increased LDE and stance time CV in elderly fallers compared to nonfallers. No significant difference in MSRS scores was found between fallers and nonfallers, therefore the relationship between reinvestment and fall history was not supported. Directing attention to the walking surface did not lead to improved gait stability in elderly, compared to internal attention on leg movement. Therefore, the possible benefits of external focus for balance control might not be present in elderly gait.

## Conflict of interest

The authors declare no conflicts of interest.
